# Anterolateral Thoracic Myelomeningocele With Split Cord Malformation

**DOI:** 10.7759/cureus.46496

**Published:** 2023-10-04

**Authors:** Jessica Ran, Preny Karamian, Zoe Robinow, Forshing Lui, David Gonda

**Affiliations:** 1 Neurosurgery, California Northstate University College of Medicine, Elk Grove, USA; 2 Clinical Sciences, California Northstate University College of Medicine, Elk Grove, USA; 3 Neurosurgery, Rady Children's Hospital-San Diego, San Diego, USA

**Keywords:** neural tube defect, thoracic meningocele, spina bifida, lateral myelomeningocele, anterolateral myelomeningocele, spinal dysraphism, thoracic myelomeningocele, split cord malformation, myelomeningocele

## Abstract

We present a case of a two-year-old male with a history of congenital scoliosis and anterolateral thoracic meningocele. He was able to walk and run, but his parents reported left leg weakness and a frequent cough. The patient had normal neurological examination findings. Magnetic resonance imaging (MRI) of the spine without contrast showed left convex upper thoracic congenital scoliosis and rightward anterolateral meningocele inferiorly to T3, with the spinal cord tethered at this location. Neurosurgical cord detethering and repair of the meningocele were performed simultaneously with scoliosis repair by orthopedics. During the dissection of the meningocele, the bulging neural tissue was found to be a split cord ending in a blind stump. The split cord was determined to be nonfunctional via Prass probe (Medtronic, Minneapolis, MN) stimulation and was subsequently dissected. Detethering of the spinal cord was followed by repair of the dural outpouching and dural closure. The patient was stable post-surgery, but long-term results remain to be seen.

## Introduction

Spina bifida is a congenital neurological disorder that results from failure of neural tube closure during fetal development [1]. During normal gastrulation, the endoderm and ectoderm interact to form the embryonic disc. The embryonic disc cells proliferate and migrate to the mesoderm where they become the trilaminar disc. The notochord and ectoderm form the neural plate, which gives rise to the neural folds and neural groove. The neural folds from either end meet and close from the cranial to the caudal end to form the neural tube [2]. The neural tube then develops into the spinal cord, brain, and meninges. However, in spina bifida, the neural tube fails to close during the 17th-30th days of fetal development, resulting in a defect of the affected vertebral level [1,2].

Spina bifida can be further classified as closed spinal dysraphism (CPD) or open spinal dysraphism (OSD). CPD has minimal neuronal involvement and may be unnoticeable or asymptomatic [1]. In OSD, the spinal defect involves neural elements that are exposed to the external environment without continuous skin coverage and can present symptomatically. Myelomeningocele (MMC) is the most severe form of spina bifida where both the meninges and spinal cord protrude out of a vertebral opening as a sac [2]. Because failure of neural tube closure most commonly occurs in the lumbosacral region, MMC is usually located in the lumbar or sacral regions and is rarer in cervical and thoracic regions [3]. The most common symptoms of MMC include loss of sensation, bladder and bowel dysfunction, renal failure, seizures, pain, and motor deficits [1,2].

In addition, lateral, anterior, or anterolateral MMC is much rarer than posterior MMC [4]. Lateral meningoceles are typically associated with neurofibromatosis type 1 or connective tissue disorders such as Marfan syndrome but may occur in isolation. Patients with lateral meningoceles may be asymptomatic or may present with paraparesis or pain due to the involvement of the spinal cord, or cough or dyspnea due to the compression of the nearby respiratory tract [5]. Occasionally, myelomeningoceles occur with split cord malformations or with tethering of the spinal cord to the skin or meninges [6-8]. Here, we present a case of a two-year-old male with a tethered right anterolateral thoracic MMC with a blind split cord malformation.

This case report was previously presented as a poster at the California Northstate University Annual Research Day on March 3, 2023.

## Case presentation

**Figure 1:**
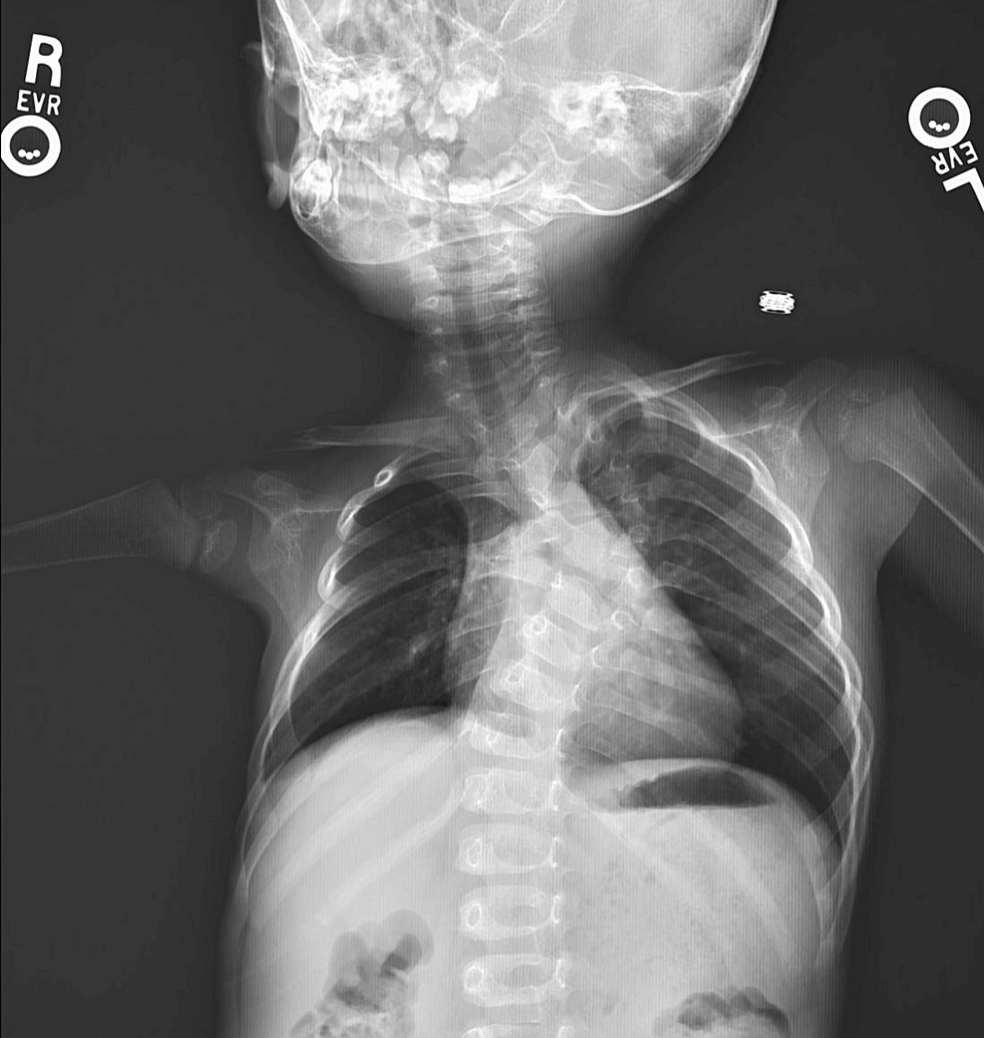
AP X-ray showing the patient's left convex upper thoracic congenital scoliosis AP: anteroposterior

**Figure 2:**
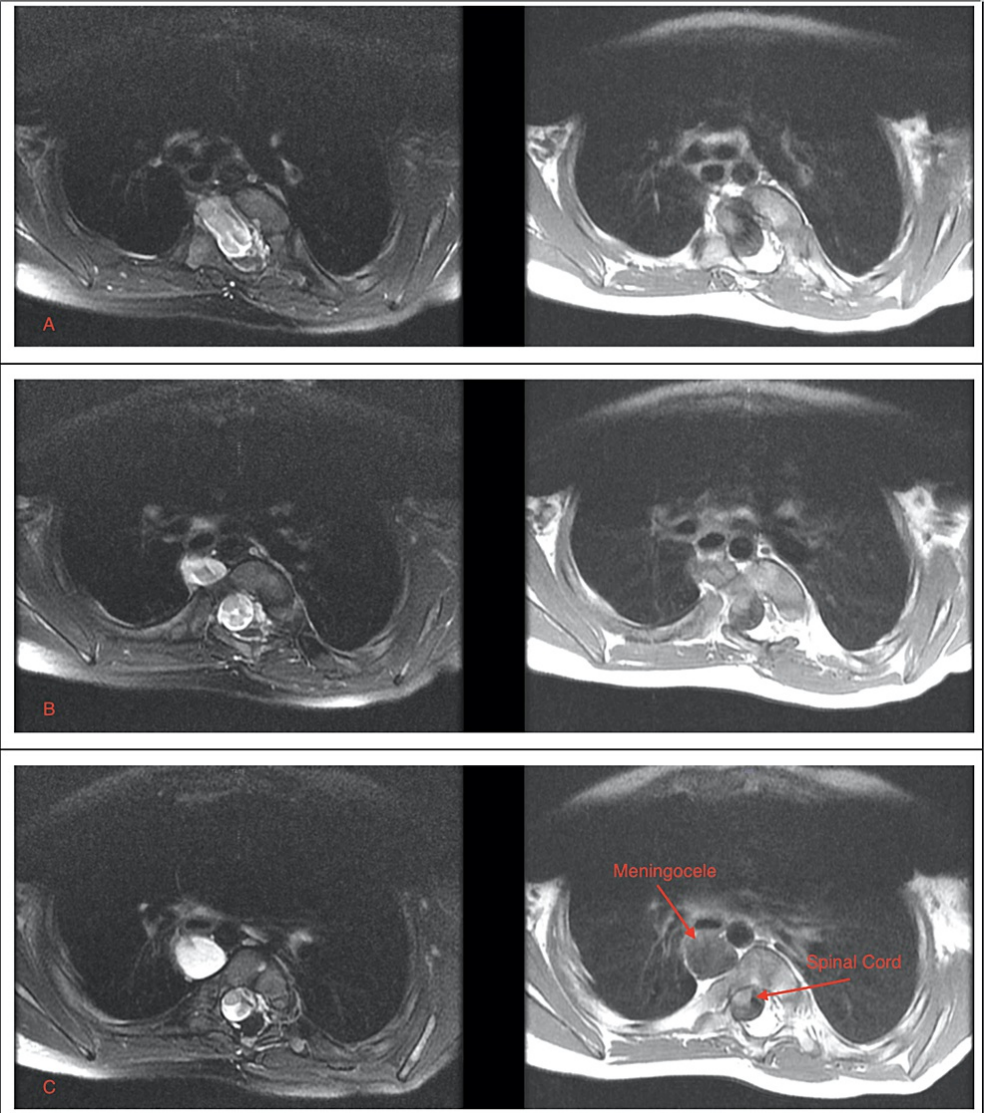
Selected axial T2-weighted and T1-weighted MRI of the thoracic spine showing the meningocele and tethered spinal cord in the vertebral column MRI: magnetic resonance imaging

**Figure 3:**
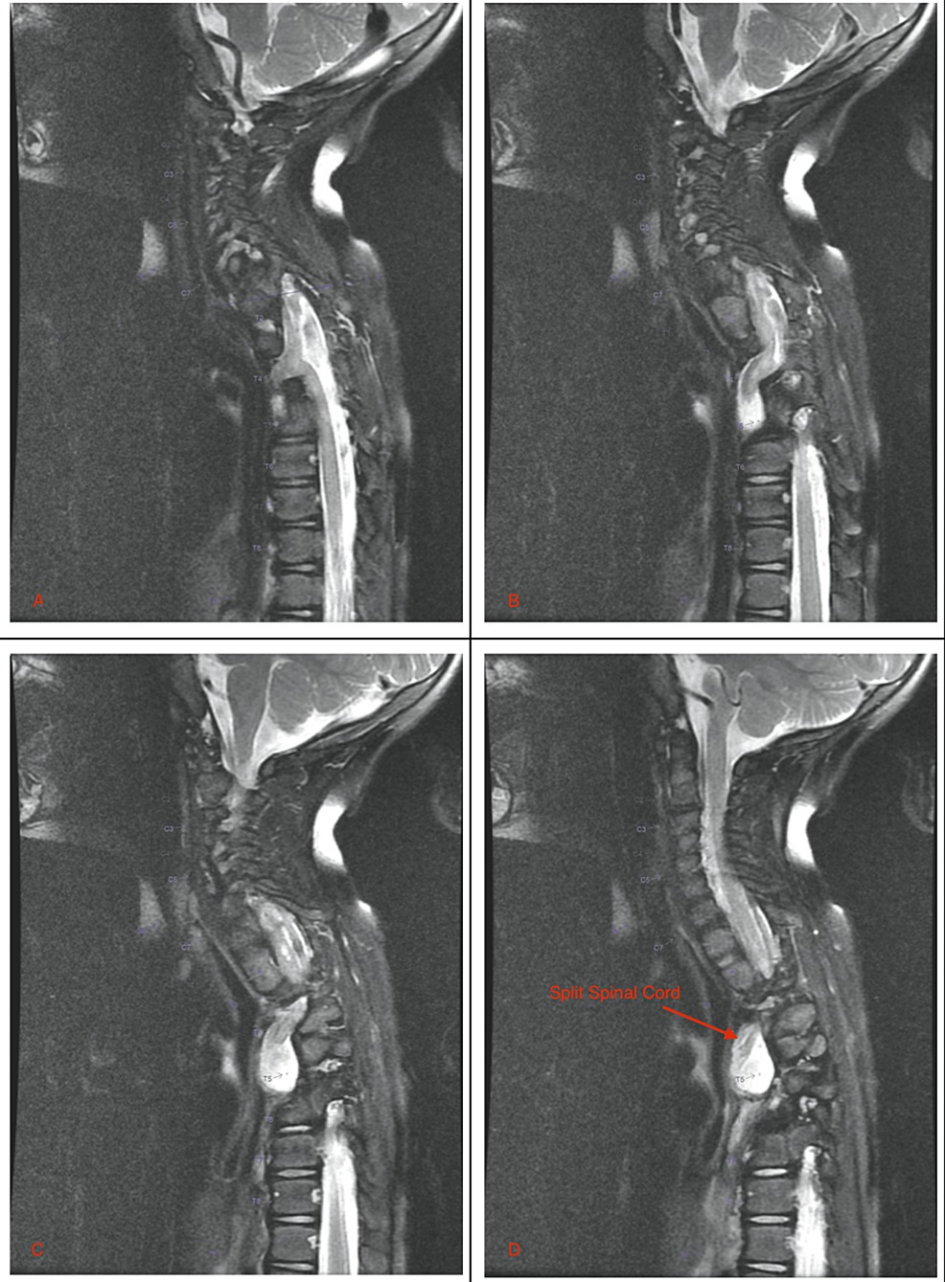
Sagittal cuts from T2-weighted MRI showing ventrally herniating myelomeningocele at T3-T4 containing CSF and spinal cord elements MRI: magnetic resonance imaging, CSF: cerebrospinal fluid

We present a case of a two-year-old male with a history of congenital scoliosis (Figure 1) and lateral thoracic meningocele. His parents reported no issues with walking or running but noted some weakness in the left leg, a frequent ongoing cough, and a tendency to use his arms to pull himself upstairs. He was born via spontaneous vaginal delivery at 39 weeks gestation and weighed 9 lb 7 oz at birth. There was no family history of brain tumors, bleeding disorders, or epilepsy. A review of systems was negative for difficulty urinating, constipation, back pain, gait problems, seizures, weakness, and headaches. On physical examination, the patient was active, playful, and neurologically intact with normal gait, motor function, and reflexes. No weakness, tremor, atrophy, clonus, abnormal muscle tone, or seizure activity was noted. Magnetic resonance imaging (MRI) of the spine without contrast showed left convex upper thoracic congenital scoliosis and a 17 mm rightward anterolateral meningocele inferiorly to T3, with the spinal cord tethered at this location (Figures 2A-2C, 3A-3D). However, it was unclear in the images whether the meningocele contained elements of the spinal cord as opposed to just meninges and cerebrospinal fluid (CSF). Additionally, at approximately T1-T2, there was a 2 mm syringohydromyelia, and at C2-C3, there was punctate prominence of the central canal.

Surgery was planned because of concern that the patient's meningocele was causing tethering and contributing to the progression of his scoliosis and left leg weakness, as well as his cough. Neurosurgical cord detethering and repair of meningocele were performed simultaneously with scoliosis repair by orthopedics. During the intradural dissection of the meningocele, the bulging neural tissue was found to be a split cord ending in a blind stump. The split cord was housed in the same dural sac, meaning this was a type 2 split cord malformation [7]. A Prass probe (Medtronic, Minneapolis, MN) was used to stimulate this segment of the spinal cord that had split from the main cord, and no stimulation was observed. Therefore, the split cord was determined to be nonfunctional, and bipolar electrocautery followed by sharp dissection was used to disconnect the spinal cord from the lateral aspect of the tethered dura. Dissection was carried down until the spinal cord was fully detethered. The presumed right T4 nerve root was sacrificed intradurally during the operation. Detethering was followed by repair of the dural outpouching. Sutures were used to close the intradural portion of the outpouching, and the dural region was covered with an intradural non-suturable graft and thereafter portions of an amniotic membrane to prevent re-tethering. Dural closure was then achieved with a small duraplasty with a dura substitute onlay. The surgery was completed with no immediate complications, and the patient was taken to the pediatric intensive care unit (PICU) in stable condition.

## Discussion

Lateral or anterior meningoceles are not as easily recognized as the more common posterior meningocele and may be asymptomatic; thus, many are discovered and treated during adulthood. There have been a few reports of lateral meningoceles presenting in early childhood as a gluteal mass [9], not seen in our patient. Our patient was not found to have features of neurofibromatosis type 1, Marfan syndrome, or lateral meningocele syndrome, which are conditions that have been associated with lateral thoracic meningoceles [5]. Additionally, almost all described anterior meningoceles have been located in the sacral region, which may manifest as urinary difficulty or other symptoms due to pressure on pelvic organs [3,4]. Our patient's myelomeningocele was located in the thoracic region and is best described as anterolateral, making it extremely unique. Notably, split cord malformations have been described coexisting with posterior myelomeningoceles [6,7] but never before with an anterior or lateral myelomeningocele. Based on currently accepted theories that split cord malformations arise due to abnormal notochord development [7], the coexistence of our patient's split cord malformation and myelomeningocele suggests an error that occurred during the third to fourth weeks of embryogenesis. Due to the absence of significant family or obstetric history and the lack of similarly described cases, it is unknown what caused our patient's neural tube defect and whether or not it may have been related to the patient's scoliosis.

Astute clinical decision-making and careful operation were crucial in the management of this case because such surgery on this type of rare neural tube defect is both medically necessary and risky; the surgery could have resulted in major complications or further injury to the patient. Long-term follow-up of the patient's symptoms is required because the patient was only two years old at the time of the operation, and it is unknown whether future complications will arise, for example, related to the sacrifice of the right T4 nerve root, or if the presenting complaints (i.e., cough and left leg weakness) will resolve.

Proper diagnosis and formulation of a treatment plan were essential, especially considering the meningocele was not visible from the exterior and the patient was considered neurologically intact on examination. MRI of the meningocele did not clearly show all of the components of the spinal defect; the split cord was discovered during surgery, and therefore, the meningocele was reconsidered as myelomeningocele as it was found to contain spinal cord elements. The use of a Prass probe, which is a stimulator probe used for nerve monitoring during surgery, illustrated the surgeon's decision-making when encountering a finding with unknown significance such as the split cord ending in a blind stump. Collaboration with orthopedics was also well executed because the patient's meningocele occurred in the setting of congenital scoliosis that needed to be repaired. We hope that this case report provides an example of a rare congenital neurological condition and the course of action taken by a neurosurgeon when presented with a novel case with high stakes.

## Conclusions

In summary, this case illustrates the diagnosis and treatment of a rare case of anterolateral thoracic myelomeningocele with a split cord malformation. Surgeons and radiologists should be aware of the possibility of this congenital anomaly. MRI and skilled surgical techniques are important for the management of both common and more novel pediatric neurosurgery cases. Because this is not a classic pediatric neurosurgery case, long-term follow-up is especially necessary to determine the outcomes of the operation.

## References

[1] Brea CM, Munakomi S (2023). Spina bifida. StatPearls [Internet].

[2] Alruwaili AA, Das JM (2023). Myelomeningocele. StatPearls [Internet].

[3] Kumar J, Afsal M, Garg A (2017). Imaging spectrum of spinal dysraphism on magnetic resonance: a pictorial review. World J Radiol.

[4] Dahlgren RM, Baron EM, Vaccaro AR (2006). Pathophysiology, diagnosis, and treatment of spinal meningoceles and arachnoid cysts. Semin Spine Surg.

[5] Ibrahim D (2023). Lateral thoracic meningocele. https://doi.org/10.53347/rID-56054.

[6] Iskandar BJ, McLaughlin C, Oakes WJ (2000). Split cord malformations in myelomeningocele patients. Br J Neurosurg.

[7] Ansari S, Nejat F, Yazdani S, Dadmehr M (2007). Split cord malformation associated with myelomeningocele. J Neurosurg.

[8] Dias MS, Wang M, Rizk EB (2021). Tethered spinal cord among individuals with myelomeningocele: an analysis of the National Spina Bifida Patient Registry. J Neurosurg Pediatr.

[9] Prasad GR, Rashmi T (2016). Lateral meningomyelocele in a neonate: a case report. J Neonatal Surg.

